# Retraction: microRNA-155 Modulates Hepatic Stellate Cell Proliferation, Apoptosis, and Cell Cycle Progression in Rats With Alcoholic Hepatitis *via* the MAPK Signaling Pathway Through Targeting SOCS1

**DOI:** 10.3389/fphar.2021.840009

**Published:** 2022-01-17

**Authors:** Frontiers Editorial Office

**Affiliations:** Frontiers Media SA, Lausanne, Switzerland

**Keywords:** microRNA-155, suppressor of cytokine signaling 1, mitogen activated protein kinase signaling pathway, alcoholic hepatitis, hepatic stellate cell, proliferation and apoptosis 3

The journal and Chief Editors retract the April 7, 2020 article cited above.

Following publication, concerns were raised regarding the validity of the data in the article. The authors failed to provide the raw data or a satisfactory explanation during the investigation, which was conducted in accordance with Frontiers’ policies. Given the concerns, and the lack of raw data, the editors no longer have confidence in the findings presented in the article.

This retraction was approved by the Chief Editors of Frontiers in Pharmacology and the Chief Executive Editor of Frontiers. The authors have not responded to any correspondence regarding the retraction.

